# Brugada-Like Electrocardiographic Pattern

**Published:** 2003-07-01

**Authors:** Johnson Francis, Charles Antzelevitch

**Affiliations:** * Associate Professor of Cardiology, Medical College Calicut, Kerala, India; † Executive Director, Masonic Medical Research Laboratory, Utica, New York, USA

The Brugada syndrome is characterized by a ST-segment elevation in the right precordial leads associated with right bundle branch block (RBBB) pattern and a propensity for life-threatening ventricular arrhythmias in the absence of structural heart disease [1]. Mutations in a cardiac sodium channel gene have been linked to this syndrome [2].

 The mechanism underlying the RBBB and ST-segment elevation in right precordial leads in patients with the Brugada syndrome is thought to be an outward shift of the ionic currents during early repolarization causing a marked accentuation of the action potential notch in right ventricular epicardial but not endocardial cells. The outward shift of current ultimately leads to loss of the action potential dome causing marked abbreviation of the action potential in the right ventricular epicardial cells [3-5].

 Local pressure applied to the right ventricular wall has also been reported to induce an ECG pattern similar to the Brugada syndrome. Tarin et al reported a patient with a mediastinal tumor and electrocardiographic findings similar to those described in the Brugada syndrome. This ECG pattern disappeared after tumor removal, thus suggesting that it was probably caused by compression of the right ventricular outflow tract by the mass [6]. Another case of pericardial fluid and "tumour" compressing the right ventricle with Brugada-like ECG pattern in a patient with rheumatoid arthritis has also been reported [7] During surgery the "tumour" was found to be organised haemopericardium. After the surgery the patient was well and had a normal ECG.

 Nakazato et al describes a similar situation in this issue of the journal [8]. Compression of the right ventricular outflow tract by an abnormal infective mass, with/without focal pericardial inflammation was thought to be the mechanism of Brugada-like ST elevation in their patient.

 The ability of local pressure to give rise to an ST segment elevation has been demonstrated experimentally in the arterially perfused right ventricular wedge preparation (Antzelevitch & Dumaine, 2002) [9]. Focal pressure was shown to cause loss of the action potential dome at some right epicardial sites but not others. The potential for this mechanism to give rise to closely coupled phase 2 reentrant extrasystoles and VT was also demonstrated in this experimental study.

Several other instances of Brugada-like patterns have been reported. Ortega-Carnicer et al [10] noted transient Brugada-type electrocardiographic abnormalities in renal failure which was reversed by dialysis. They reported a patient with a previous history of epilepsy treated with psychotropic drugs (with a sodium channel blocking effect) and chronic renal failure on haemodialysis who developed hyperkalaemia and ECG findings resembling Brugada syndrome. These ECG changes disappeared after haemodialysis when the potassium became normal. They concluded that hyperkalaemia along with cardiac membrane active drugs may cause ECG changes mimicking the Brugada syndrome.

 Transient Brugada pattern has been observed repeatedly after recreational use of cocaine [11]. Intravenous administration of procainamide and subsequent intravenous propranolol followed by noradrenaline failed to reproduce the Brugada in this case. Electrophysiologic study performed in the presence of the Brugada ECG pattern showed no inducible arrhythmias. Yet another report describes a patient in whom a typical Brugada ECG pattern developed in relation to fever but could not be reproduced at normal temperature on administration of flecainide [12]. This case suggests that in some patients a Brugada-like ECG may only manifest during a febrile state.

All these reports of Brugada-like ECG pattern give us a better insight into the genesis of this pattern and possibly localize the abnormality to the right ventricular outflow tract. Heterogenous response of repolarization across the ventricular wall in the right ventricular outflow tract is thought to be responsible for accentuation of ST segment elevation in the right precordial leads [13].
